# Pharmacological diacylglycerol lipase inhibition impairs contextual fear extinction in mice

**DOI:** 10.1007/s00213-023-06523-3

**Published:** 2024-01-06

**Authors:** Liorimar Ramos-Medina, Luis E. Rosas-Vidal, Sachin Patel

**Affiliations:** 1 Vanderbilt Brain Institute, Vanderbilt University, Nashville, TN 37232 USA; 2 Northwestern Center for Psychiatric Neuroscience, Department of Psychiatry and Behavioral Sciences, Northwestern University Feinberg School of Medicine, Chicago, IL 60611 USA

**Keywords:** 2-AG, Extinction, Mice, Fear, Cannabinoid, Stress, Learning, Memory

## Abstract

Acquisition and extinction of associative fear memories are critical for guiding adaptive behavioral responses to environmental threats, and dysregulation of these processes is thought to represent important neurobehavioral substrates of trauma and stress-related disorders including posttraumatic stress disorder (PTSD). Endogenous cannabinoid (eCB) signaling has been heavily implicated in the extinction of aversive fear memories and we have recently shown that pharmacological inhibition of 2-arachidonoylglycerol (2-AG) synthesis, a major eCB regulating synaptic suppression, impairs fear extinction in an auditory cue conditioning paradigm. Despite these data, the role of 2-AG signaling in contextual fear conditioning is not well understood. Here, we show that systemic pharmacological blockade of diacylglycerol lipase, the rate-limiting enzyme catalyzing in the synthesis of 2-AG, enhances contextual fear learning and impairs within-session extinction. In sham-conditioned mice, 2-AG synthesis inhibition causes a small increase in unconditioned freezing behavior. No effects of 2-AG synthesis inhibition were noted in the Elevated Plus Maze in mice tested after fear extinction. These data provide support for 2-AG signaling in the suppression of contextual fear learning and the expression of within-session extinction of contextual fear memories.

## Introduction

During exposure to a traumatic or aversive event, fear subserves an evolutionary conserved function across mammals: for the individual to learn the relevant information about threat-predictive environments to avoid harm (Davis et al. 2010). Contextual and discrete cues and the traumatic event become associated when present in close temporal or spatial proximity (contiguity) and if they reliably predict the occurrence of the aversive event (contingency). This process is termed associative learning and it enables organisms to acquire information about relationships between various environmental events (Takehara-Nishiuchi 2022) and facilitates appropriate selection of fear responses (Kozlowska et al. 2015). However, in trauma-related disorders, patients exhibit dysregulation of physiological associative learning processes, including impaired extinction of conditioned fear responses, impaired recall of extinction once learned, and/or stimulus generalization (APA 2013; Difede et al. 2014; Mahan and Ressler 2012; Milad et al. 2009; Milad and Quirk 2012). This compromises the ability of the individual to perform adaptatively in novel, safe contexts (Goode and Maren 2019).

 The study of conditioned fear in animal models is often based on the associative relationship between an unconditioned stimulus (US), such as an electric footshock, with a neutral stimulus, like a particular sound, context, or odor (LeDoux 2000; Pavlov 1927). The neutral stimulus acquires aversive properties and becomes a conditioned stimulus (CS) after being paired contingently with the US. On the other hand, extinction of conditioned fear requires the repeated presentation of the context or cues associated with the traumatic event, but without the aversive consequences (Milad and Quirk 2012). While the original conditioned fear memory remains within the brain and ready to emerge under specific conditions (Bouton 1994; Lacagnina et al. 2019; Maren and Quirk 2004), extinction entails new associative learning in which the context and discrete cues associated with the trauma are now rendered safe, resulting in a decrease of the conditioned fear response (Quirk 2002; Sierra-Mercado et al. 2011). Importantly, fear extinction is thought to be orchestrated by separate cell engrams than those recruited for fear learning (Lee and Kaang 2023), with three temporally distinct stages: acquisition, consolidation, and retrieval (Quirk and Mueller 2008). Each stage of extinction learning possesses a particular molecular signature (Cestari et al. 2014; Herry and Mons 2004), and can be modulated by distinct neurotransmitter signaling systems (Bouton et al. 2021; Furini et al. 2014).

Previous research has implicated the cannabinoid signaling system in the learning of extinction, both in human subjects and in rodent models of fear (Gunduz-Cinar et al. 2013; Hammoud et al. 2019; Marsicano et al. 2002; Mayo et al. 2020; Rabinak et al. 2014; Rabinak et al. 2013). Of particular interest are endogenous cannabinoids (eCB) such as 2-Arachydonoylglycerol (2-AG), the most abundant eCB in the brain. 2-AG acts as a retrograde modulator of presynaptic neurotransmitter release via activation of type 1 cannabinoid receptors (Kano et al. 2009) and is synthesized by the enzyme DAGLα located in postsynaptic dendrites and spines (Tanimura et al. 2010). Recent data suggests 2-AG is recruited for extinction driven by auditory (Cavener et al. 2018; Jenniches et al. 2016) and contextual (Segev et al. 2018) cues, as well as for the integration of new spatial information (Kishimoto et al. 2015; Schurman et al. 2019). However, the role of 2-AG signaling in the learning and extinction of contextual conditioned fear has not been conclusively parsed out. Towards this end, we employed a pharmacological approach coupled with an extended context fear conditioning and extinction paradigm to study the effects of 2-AG depletion on contextual fear conditioning and extinction in both male and female C57Bl/6j mice.

## Materials and methods

### Animals

Male and female C57BL/6J mice (The Jackson Laboratory, Maine) aged ~10 weeks were used for all behavior experiments. Mice were group housed (5 mice per cage) in climate-controlled colony rooms, maintained at a temperature of 21 ± 2 °C, 30% ± 10% relative humidity. All behavior experiments were conducted during the light phase; on a 12L:12D cycle, with lights on at 0600 h. Food and water were provided ad libitum (LabDiet 5001; LabDiet). All studies were carried out in accordance with the National Institute of Health Guide for the Care and Use of Laboratory Animals and approved by the Vanderbilt and Northwestern University Institutional Animal Care and Use Committee (#M1600213-01 and IS00019672, respectively). We chose to use randomly cycling female mice across all behavioral experiments in the context of recent data demonstrating hormones do not inherently confer more variability to behavioral data due to estrous cycle (Gruene et al. 2015; Kaluve et al. 2022; Prendergast et al. 2014; Shansky 2019).

### Drug treatment

DO34 (50 mg/kg, Glixx Laboratories Inc., MA), a pharmacological inhibitor of the DAGL enzyme, the rate-limiting step of 2-AG biosynthesis (Ogasawara et al. 2016), was used throughout all experiments. It was dissolved in an 18:1:1 solution of saline (Hospira, IL, USA); ethanol (Pharmco, KY, USA); and kolliphor EL (Sigma–Aldrich, St. Louis, MO, USA). Vehicle treatment consisted of an 18:1:1 solution of saline, ethanol, and kolliphor EL. Both treatments were given at a volume of 10 ml/kg. A dose of 50 mg/kg was administered as previously described (Cavener et al. 2018) and which has been reported to cause a near-complete reduction of measurable 2-AG throughout the brain (Bluett et al. 2017; Ogasawara et al. 2016). This is the standard dose used to assess the behavioral effects of systemic 2-AG depletion across a wide range of tests. DO34 was administered through the intraperitoneal (IP) route 2 h before behavioral testing for each stage of the experimental protocol.

### Fear conditioning and extinction paradigm

**Fig. 1:**
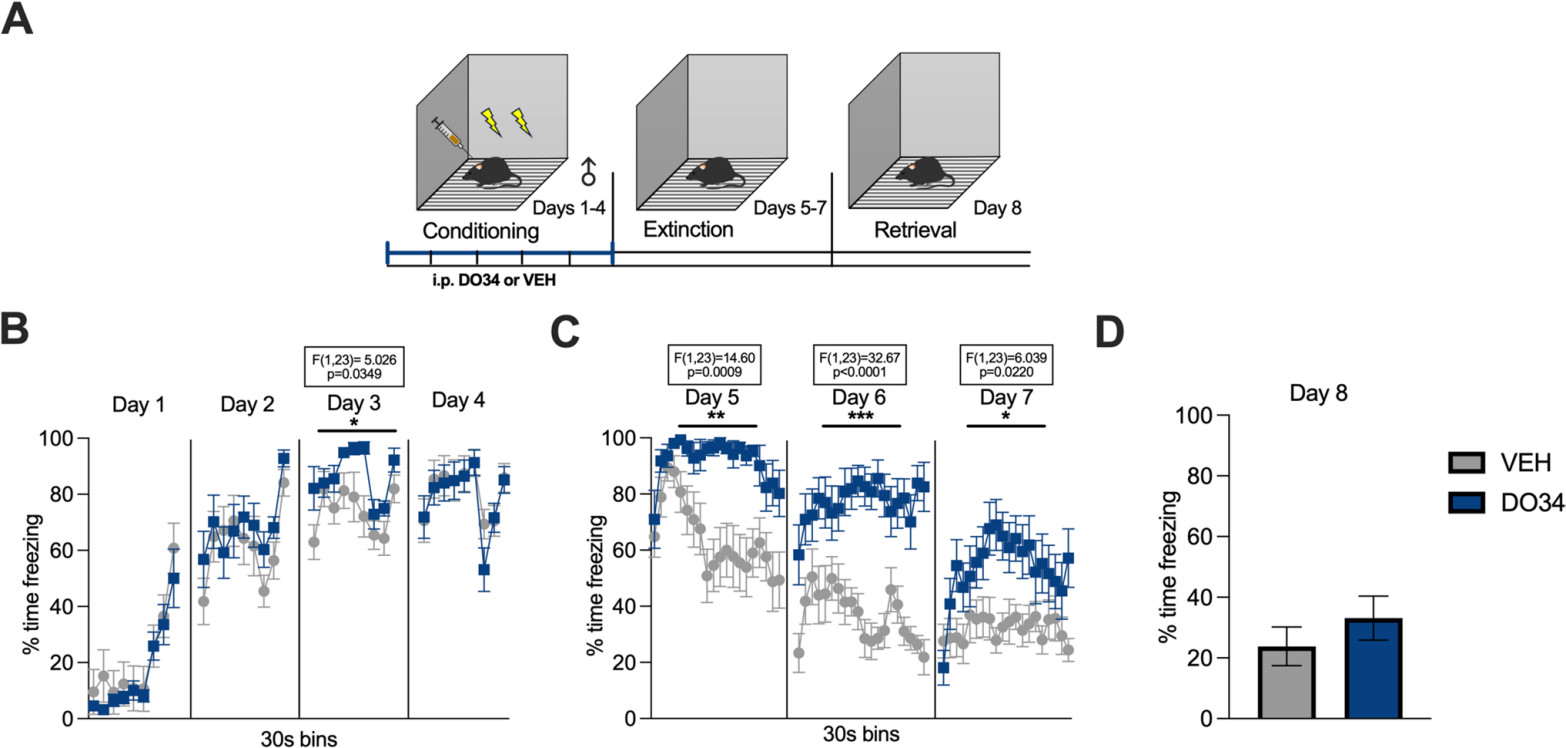
Pharmacological inhibition of DAGL during conditioning enhances fear learning and impairs within-session extinction learning in male mice. (**A**) Schematic diagram of the experimental paradigm. (**B**) Percentage of time spent freezing during the acquisition of contextual fear when DO34 (50 mg/kg) was injected IP 2 h before each session. (**C**) Percentage of time spent freezing during extinction of contextual fear in the absence of drug treatment. (**D**) Percentage of time spent freezing during retrieval session. All values are presented as mean ± SEM (*n*=12–13 male mice per condition)

**Fig. 2:**
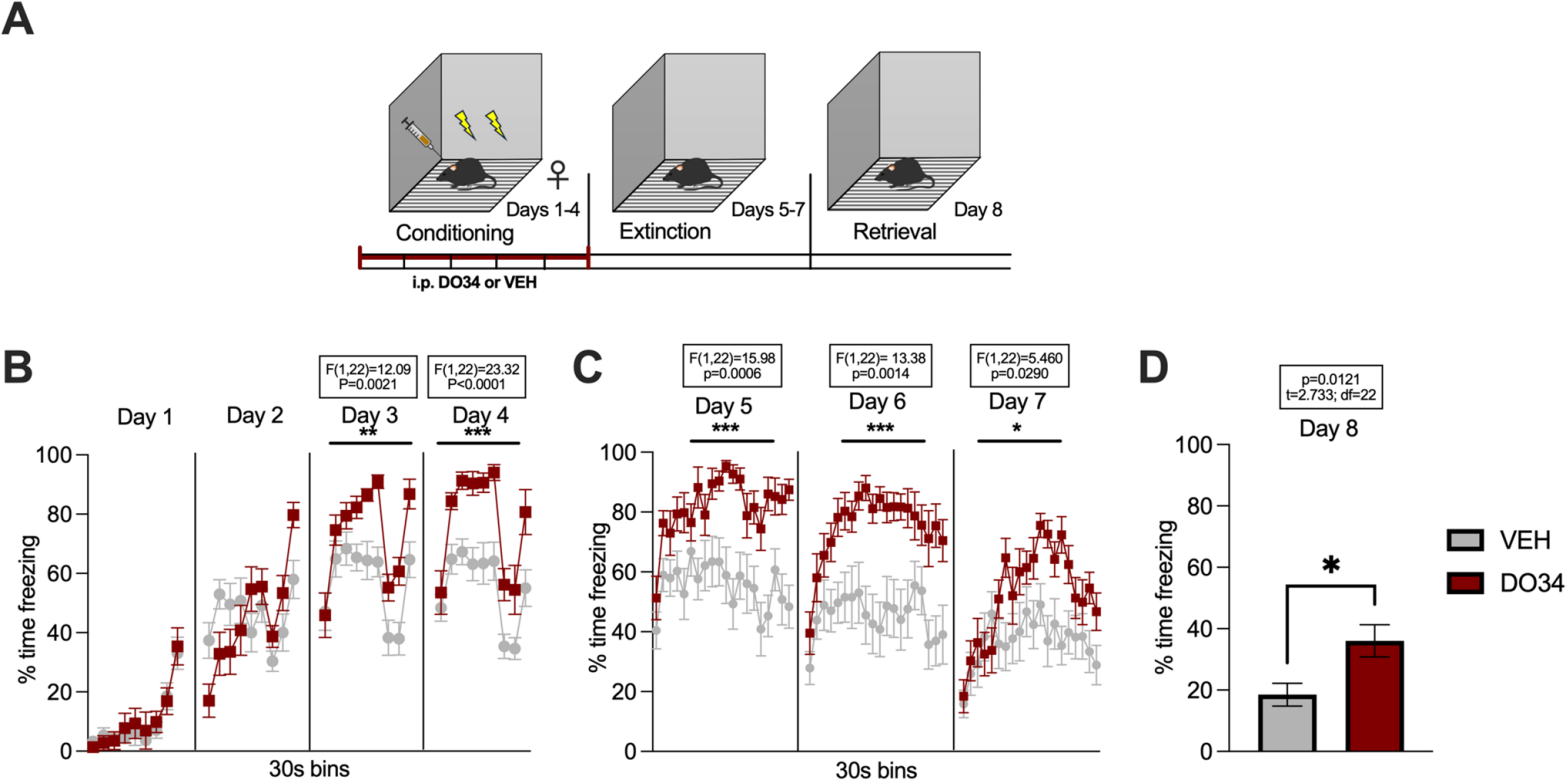
Pharmacological inhibition of DAGL during conditioning enhances fear learning and impairs within-session extinction learning and extinction retrieval in female mice. (**A**) Schematic diagram of the experimental paradigm. (**B**) Percentage of time spent freezing during the acquisition of contextual fear when DO34 (50 mg/kg) was injected IP 2 h before each session. (**C**) Percentage of time spent freezing during extinction of contextual fear in the absence of drug treatment. (**D**) Percentage of time spent freezing during retrieval session. All values are presented as mean ± SEM (*n*=12 female mice per condition)

**Fig. 3:**
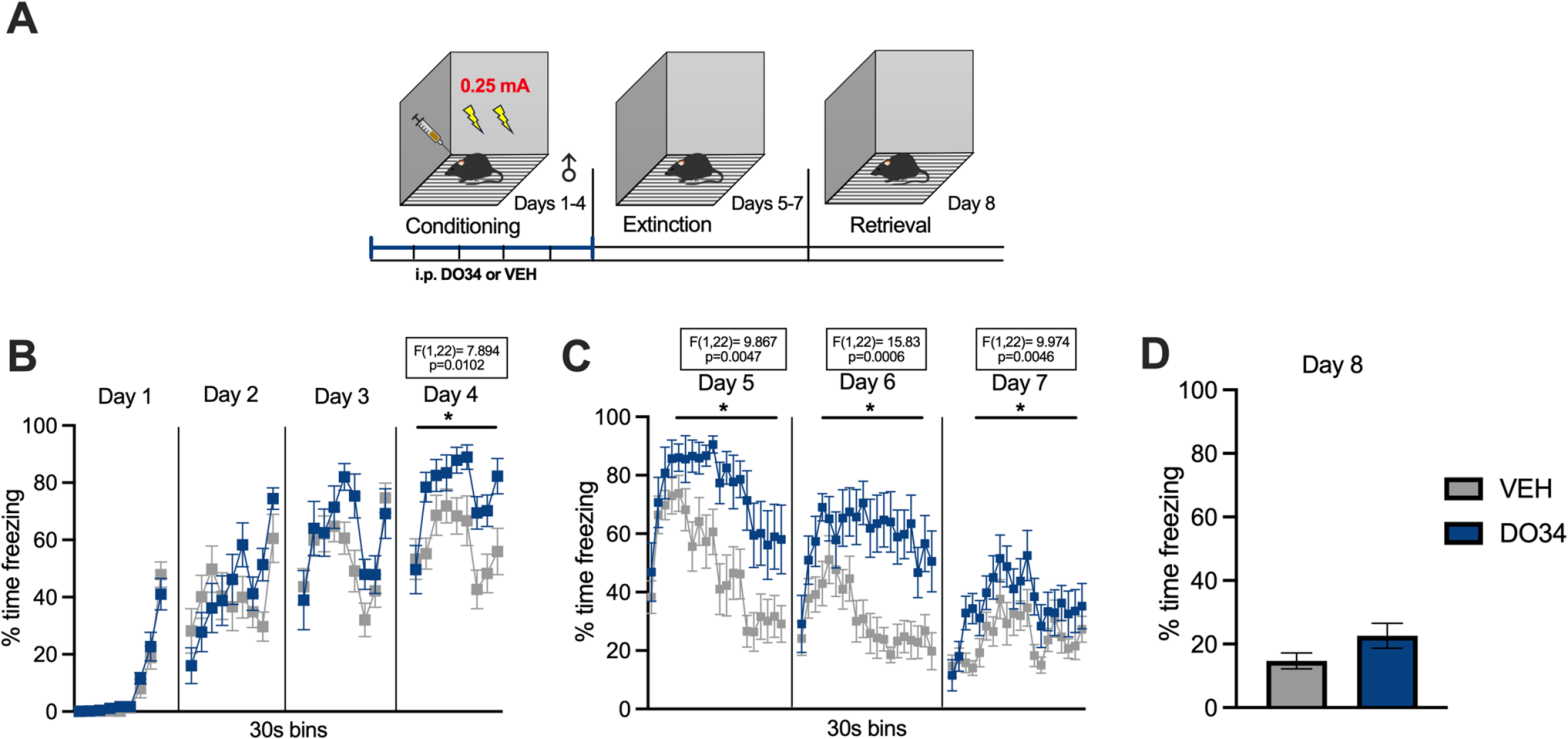
Pharmacological inhibition of DAGL during submaximal conditioning enhances fear learning and impairs within-session learning of extinction in male mice. (**A**) Schematic diagram of the experimental paradigm. (**B**) Percentage of time spent freezing during the acquisition of contextual fear when DO34 (50 mg/kg) was injected IP 2 h before each session. (**C**) Percentage of time spent freezing during extinction of contextual fear in the absence of drug treatment. (**D**) Percentage of time spent freezing during retrieval session. All values are presented as mean ± SEM (*n*=12 male mice per condition)

**Fig. 4:**
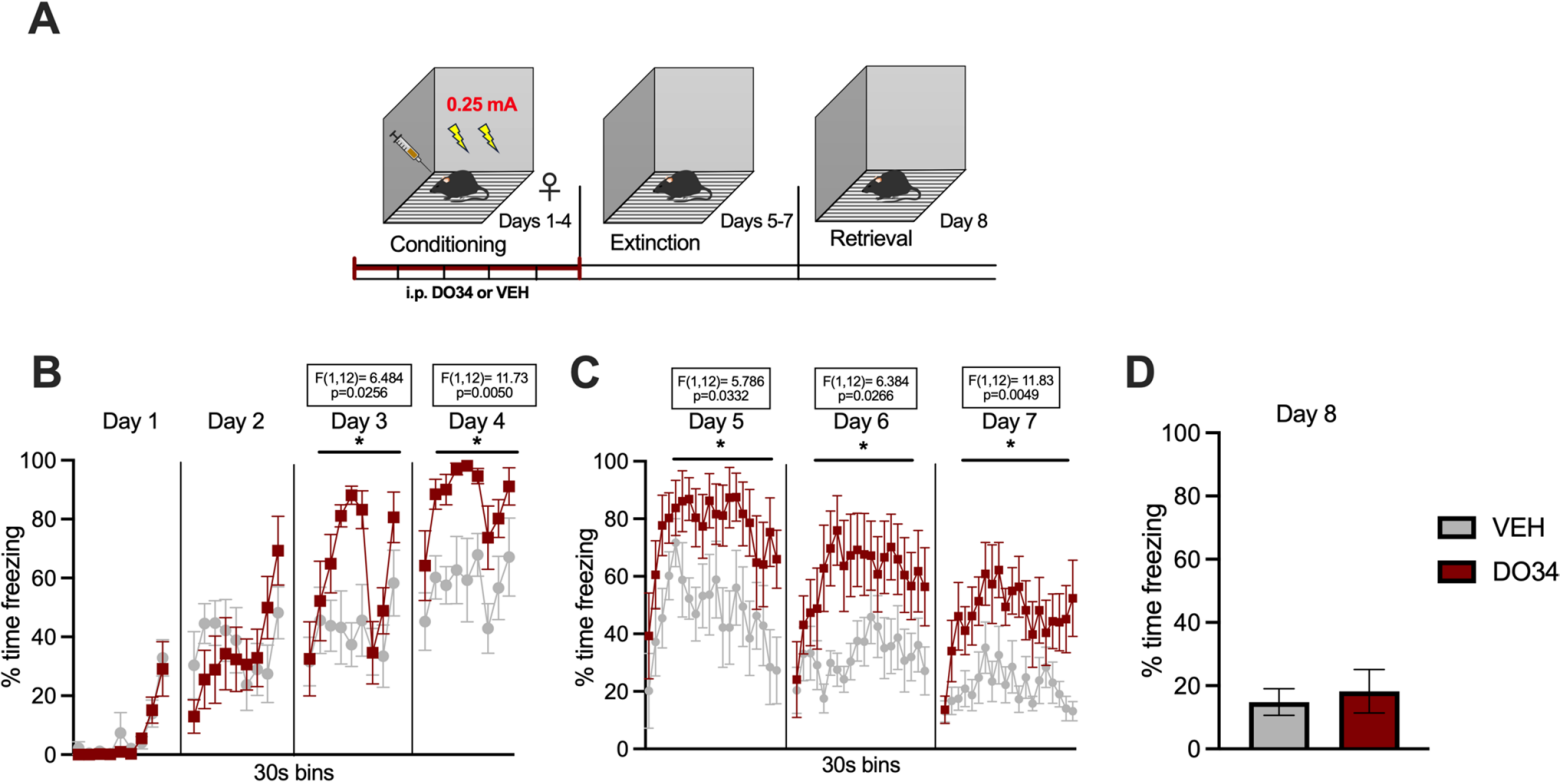
Pharmacological inhibition of DAGL during submaximal conditioning enhances fear learning and impairs within-session learning of extinction in female mice. (**A**) Schematic diagram of the experimental paradigm. (**B**) Percentage of time spent freezing during the acquisition of contextual fear when DO34 (50 mg/kg) was injected IP 2 h before each session. (**C**) Percentage of time spent freezing during extinction of contextual fear in the absence of drug treatment. (**D**) Percentage of time spent freezing during retrieval session. All values are presented as mean ± SEM (*n*=7 female mice per condition)

**Fig. 5:**
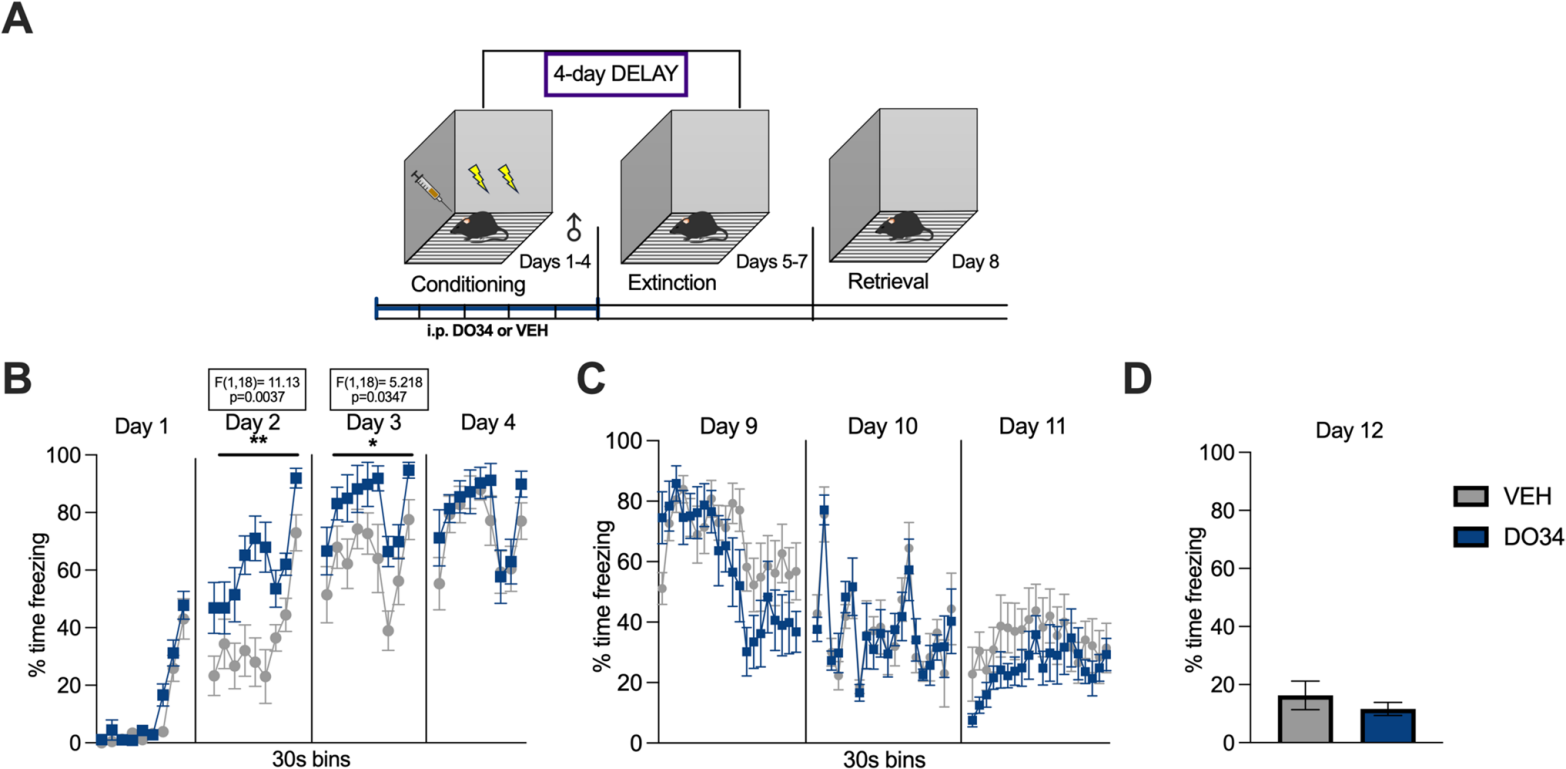
Pharmacological inhibition of DAGL during conditioning followed by a 4-day delay restores subsequent extinction learning in male mice. (**A**) Schematic diagram of the experimental paradigm. (**B**) Percentage of time spent freezing during the acquisition of contextual fear when DO34 (50 mg/kg) was injected IP 2 h before each session. (**C**) Percentage of time spent freezing during extinction of contextual fear in the absence of drug treatment. (**D**) Percentage of time spent freezing during retrieval session. All values are presented as mean ± SEM (*n*= 10 male mice per condition)

**Fig. 6:**
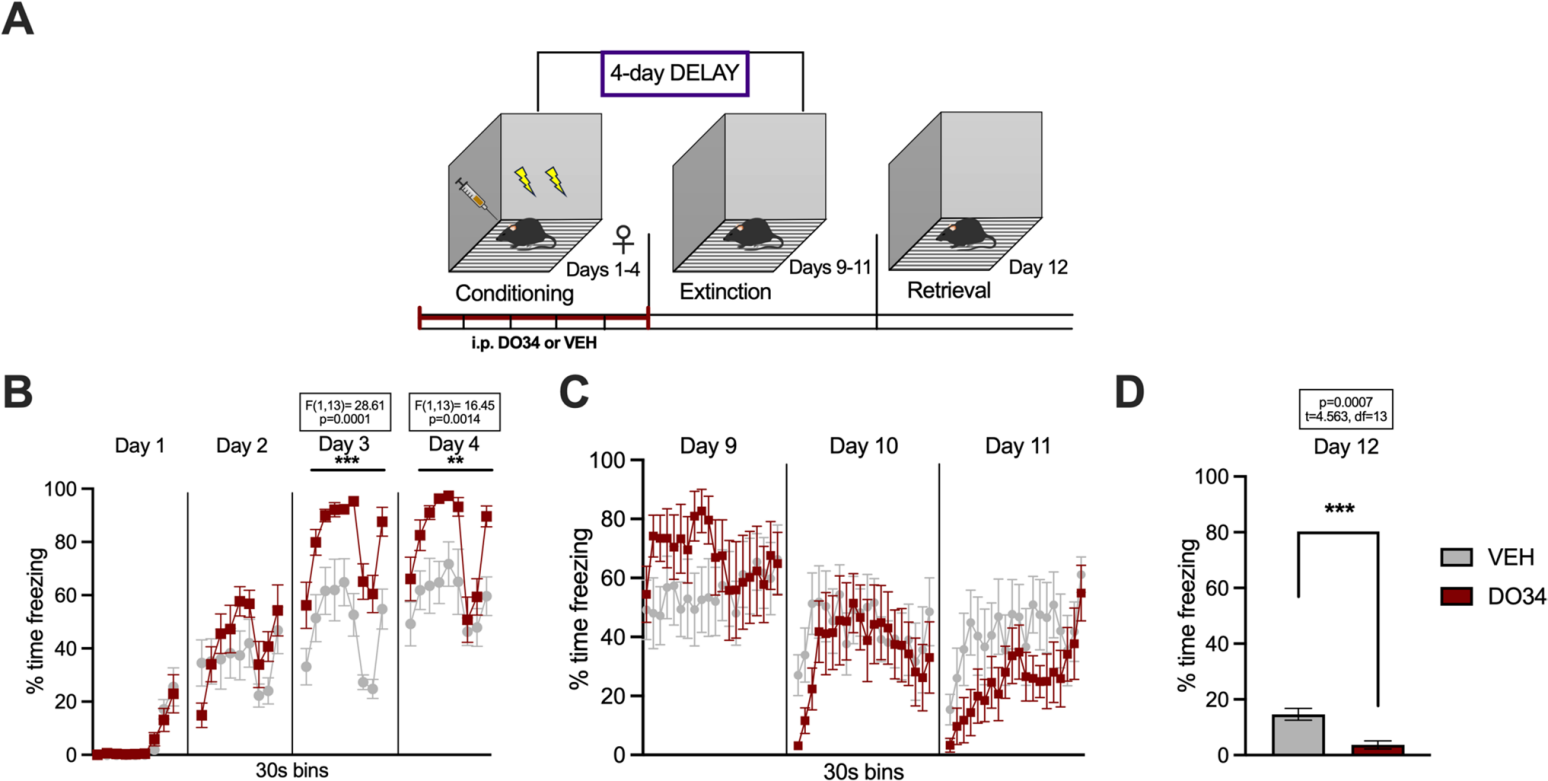
Pharmacological inhibition of DAGL during conditioning followed by a 4-day delay restores subsequent extinction learning in female mice. (**A**) Schematic diagram of the experimental paradigm. (**B**) Percentage of time spent freezing during the acquisition of contextual fear when DO34 (50 mg/kg) was injected IP 2 h before each session. (**C**) Percentage of time spent freezing during extinction of contextual fear in the absence of drug treatment. (**D**) Percentage of time spent freezing during retrieval session. All values are presented as mean ± SEM (*n*=7–8 female mice per condition)

**Fig. 7:**
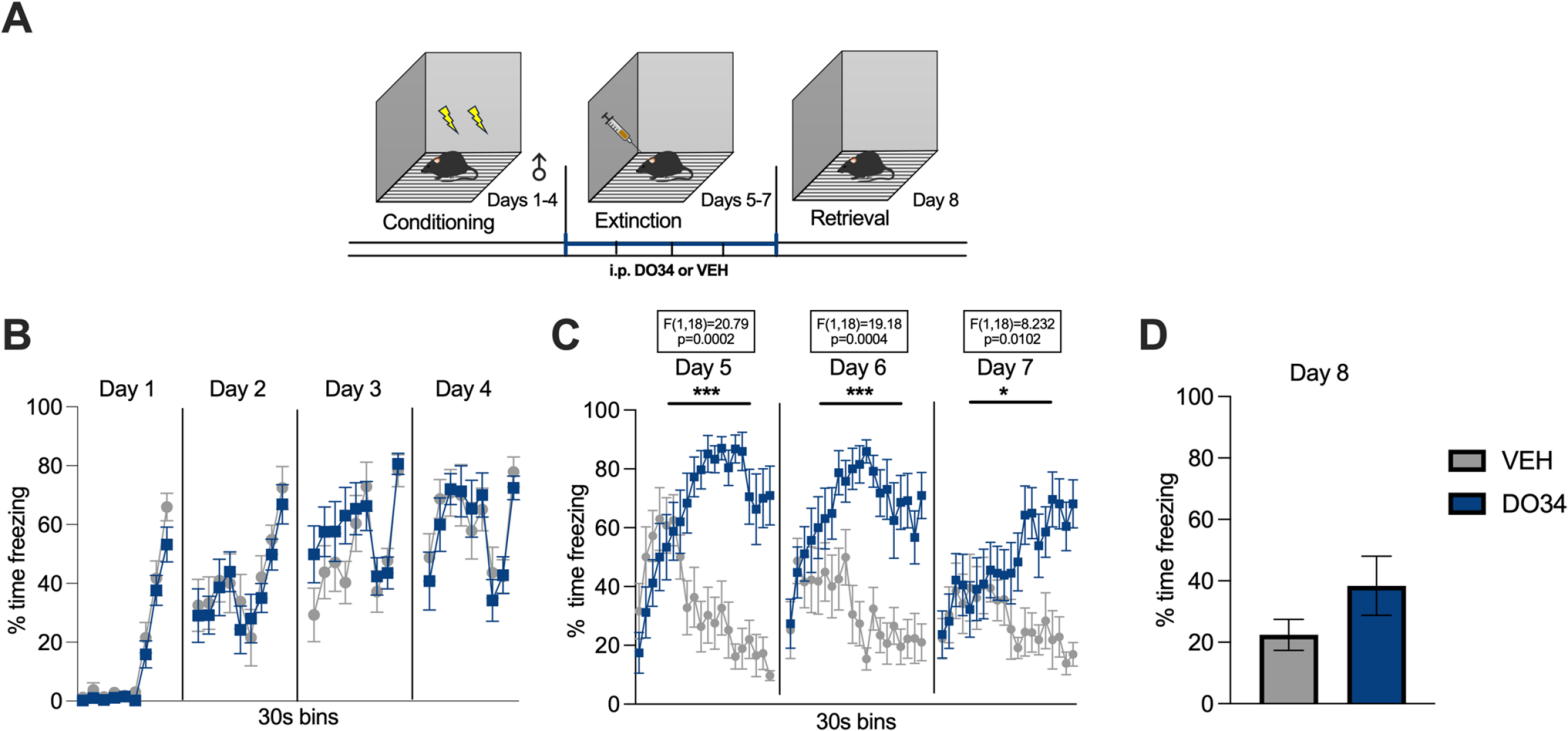
Pharmacological inhibition of DAGL during extinction training impairs within-session learning of extinction in male mice. (**A**) Schematic diagram of the experimental paradigm. (**B**) Percentage of time spent freezing during the acquisition of contextual fear. (**C**) Percentage of time spent freezing during extinction of contextual fear when DO34 (50 mg/kg) was injected IP 2 h before each extinction training session. (**D**) Percentage of time spent freezing during retrieval test. All values are presented as mean ± SEM (*n*= 10 male mice per condition)

**Fig. 8:**
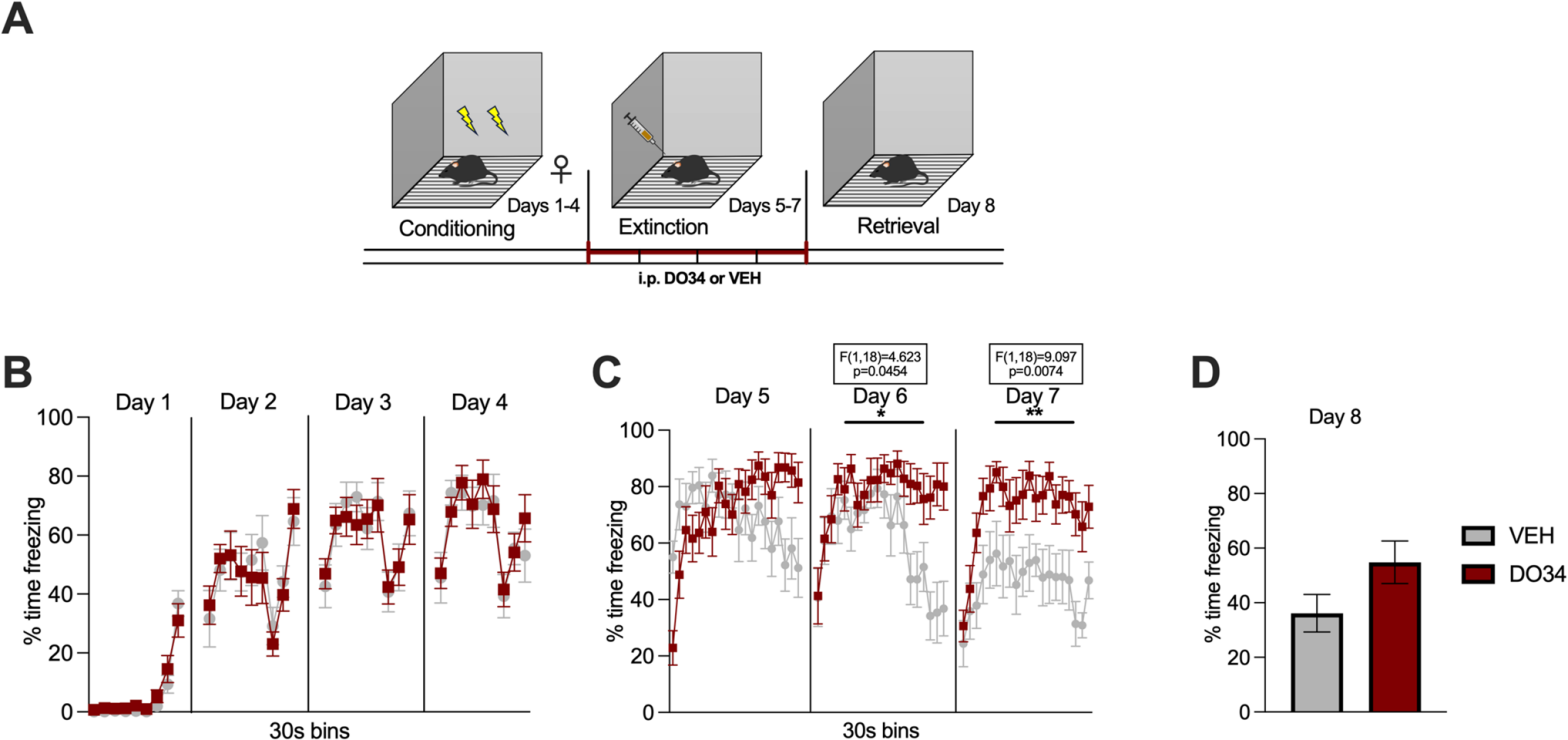
Pharmacological inhibition of DAGL during extinction training impairs within-session learning of extinction in female mice. (**A**) Schematic diagram of the experimental paradigm. (**B**) Percentage of time spent freezing during the acquisition of contextual fear. (**C**) Percentage of time spent freezing during extinction of contextual fear when DO34 (50 mg/kg) was injected IP 2 h before each extinction training session. (**D**) Percentage of time spent freezing during retrieval session. All values are presented as mean ± SEM (*n*= 10 female mice per condition).

**Fig. 9:**
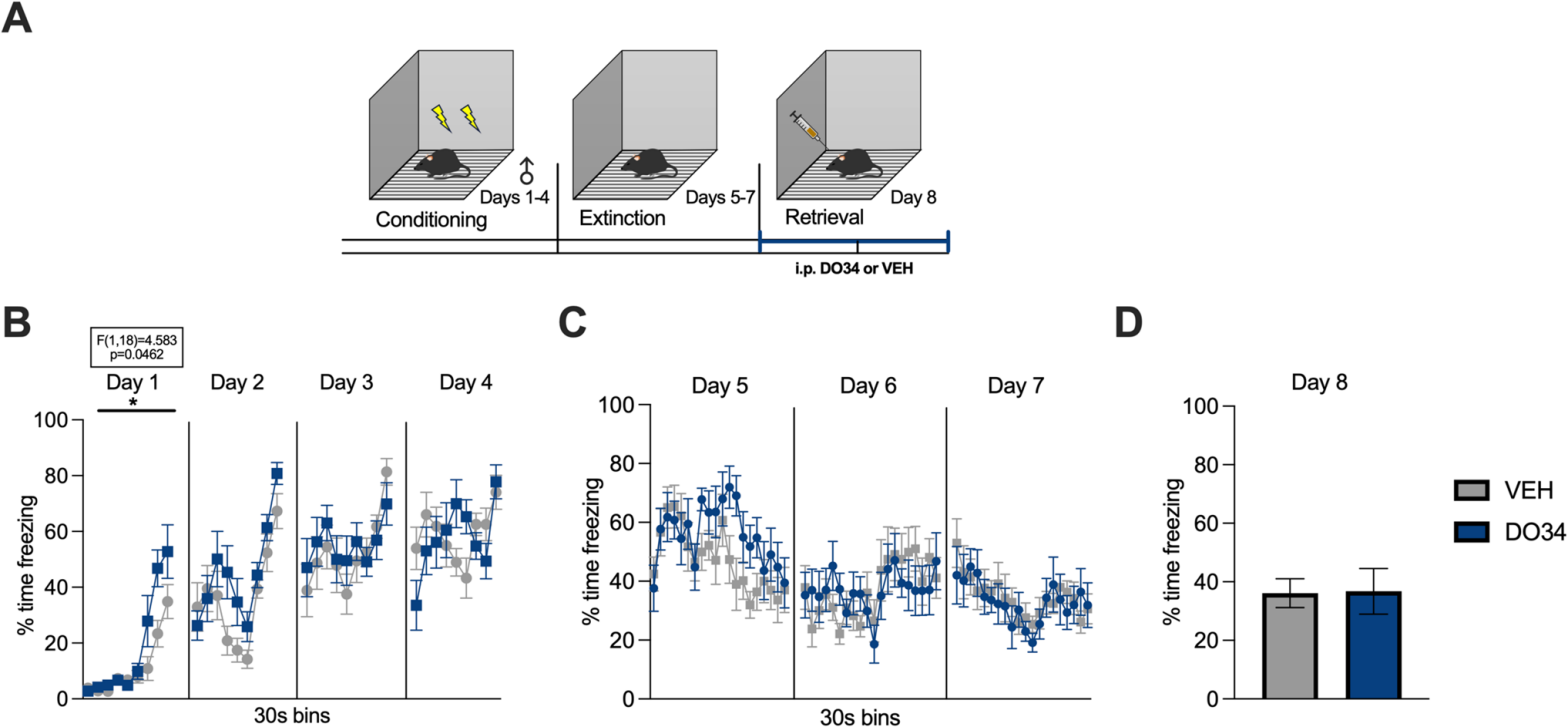
Pharmacological inhibition of DAGL during extinction retrieval does not affect the freezing behavior in male mice. (**A**) Schematic diagram of the experimental paradigm. (**B**) Percentage of time spent freezing during the acquisition of contextual fear. (**C**) Percentage of time spent freezing during extinction of contextual fear. (**D**) Percentage of time spent freezing during retrieval test when DO34 (50 mg/kg) was injected IP 2 h before each session. All values are presented as mean ± SEM (*n*= 10 male mice per condition)

**Fig. 10:**
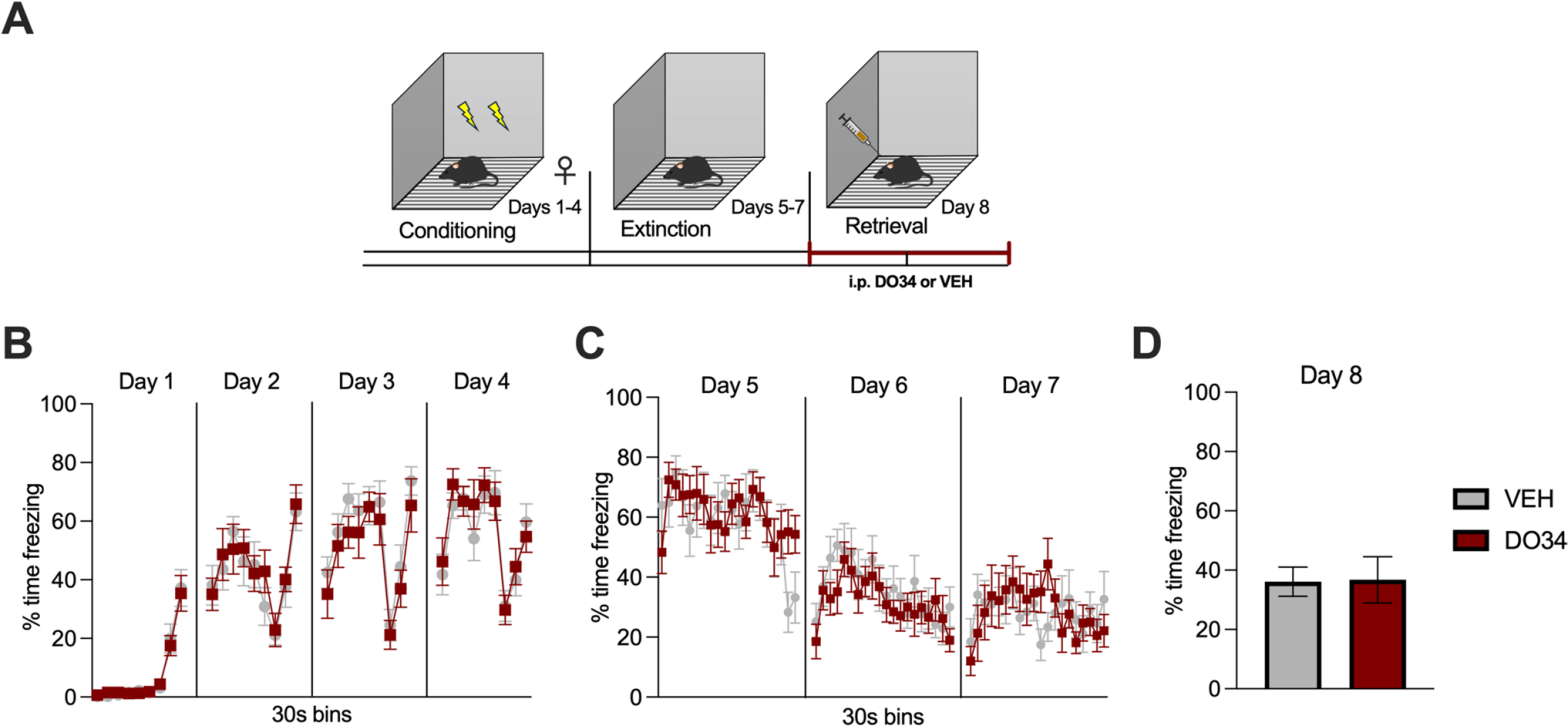
Pharmacological inhibition of DAGL during extinction retrieval does not affect the freezing behavior in female mice. (**A**) Schematic diagram of the experimental paradigm. (**B**) Percentage of time spent freezing during the acquisition of contextual fear. (**C**) Percentage of time spent freezing during extinction of contextual fear. (**D**) Percentage of time spent freezing during retrieval session when DO34 (50 mg/kg) was injected IP 2 h before each session. All values are presented as mean ± SEM (*n*= 9–10 female mice per condition)

**Fig. 11:**
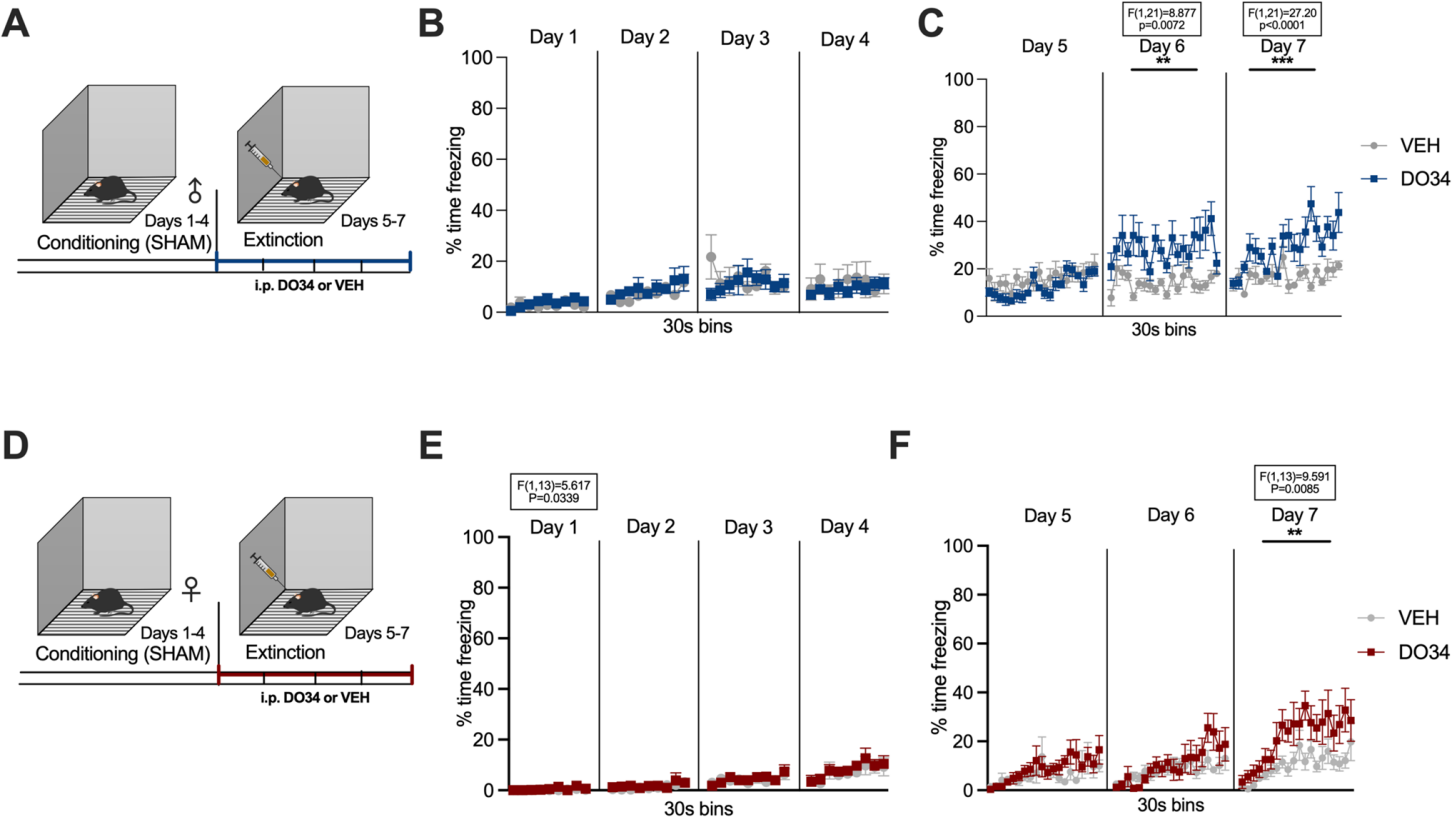
Pharmacological inhibition of DAGL during sham extinction promotes freezing in male and female mice. (**A**) Schematic diagram of the experimental paradigm in male mice. (**B**) Percentage of time spent freezing during sham-conditioning sessions. (**C**) Percentage of time spent freezing during sham extinction when DO34 (50 mg/kg) was injected IP 2 h before trial. (**D**) Schematic diagram of the experimental paradigm in female mice. (**E**) Percentage of time spent freezing during sham-conditioning sessions. (**F**) Percentage of time spent freezing during sham extinction when DO34 (50 mg/kg) was injected IP 2 h before each session. All values are presented as mean ± SEM (*n*= 10–13 male and 7–8 female mice per condition)

**Fig. 12:**
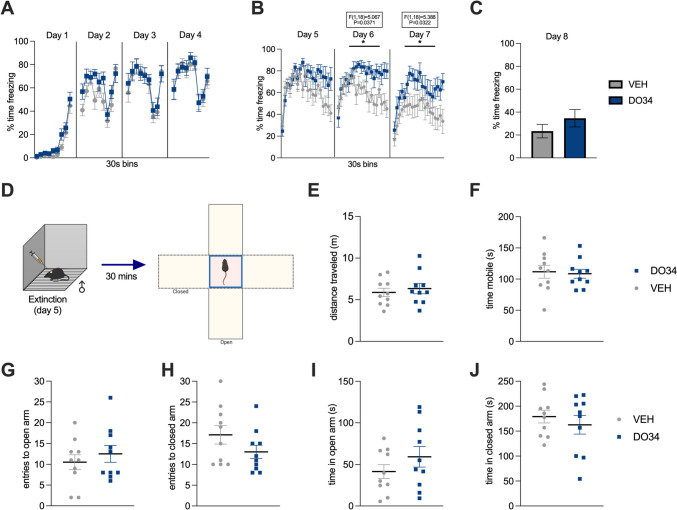
Pharmacological inhibition of DAGL during extinction does not promote anxiety-like behavior in the EPM in male mice. (**A**) Percentage of time spent freezing during acquisition of contextual fear. (**B**) Percentage of time spent freezing during extinction when DO34 (50 mg/kg) was injected IP 2 h before each session. (**C**) Percentage of time spent freezing during retrieval test. (**D**) Schematic diagram of behavioral testing for the EPM 30 min after the first extinction session. (**E**) Distance traveled in the whole apparatus. (**F**) Total immobility time. (**G**) Entries and (**H**) time spent in open arms of the maze. (**I**) Entries and (**K**) time spent in closed arms of the maze. All values are presented as mean ± SEM (*n*= 10 male mice per condition)

**Fig. 13:**
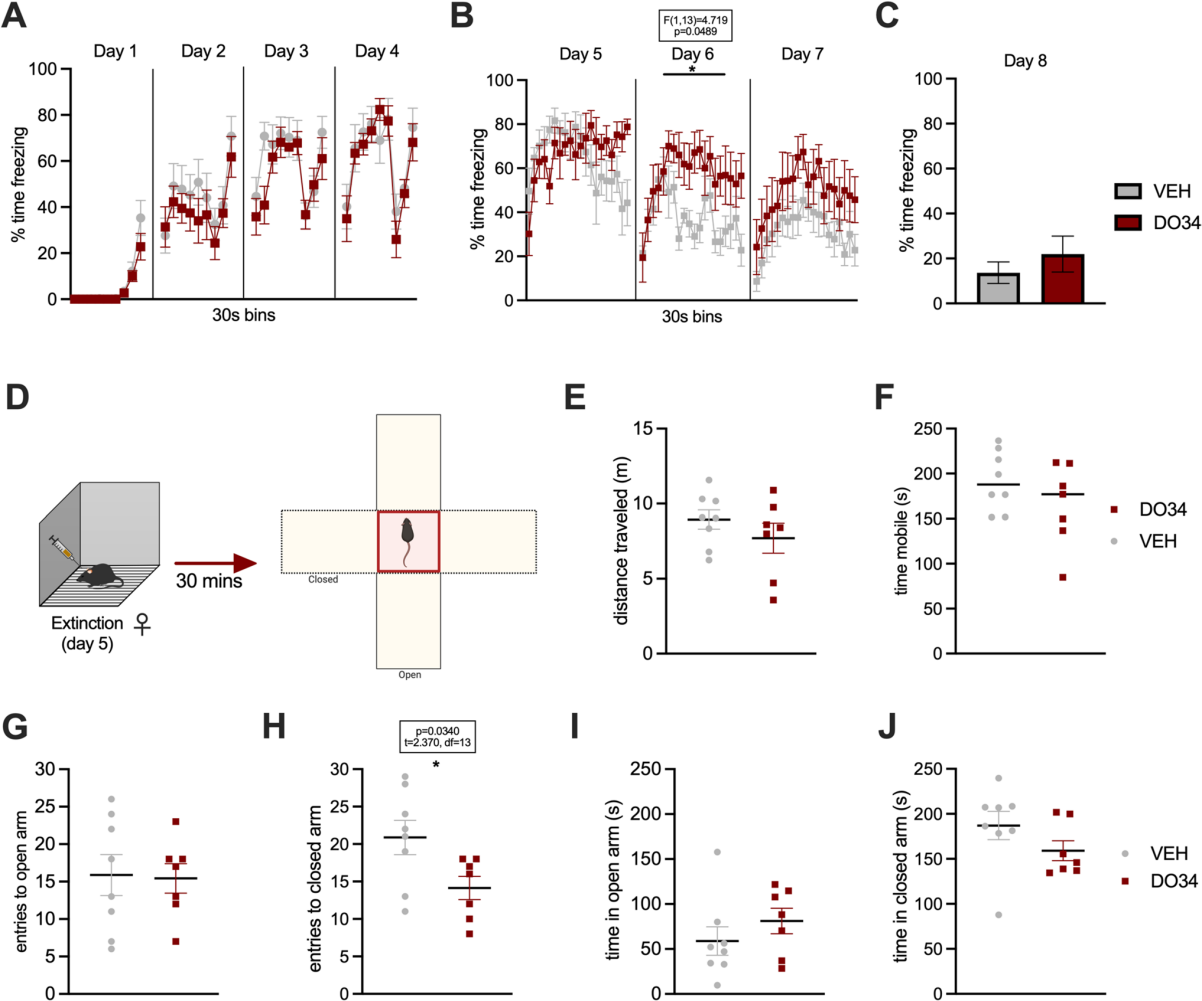
Pharmacological inhibition of DAGL during extinction does not promote anxiety-like behavior in the EPM in female mice. (**A**) Percentage of time spent freezing during acquisition of contextual fear. (**B**) Percentage of time spent freezing during extinction when DO34 (50 mg/kg) was injected IP 2 h before each session. (**C**) Percentage of time spent freezing during retrieval test. (**D**) Schematic diagram of behavioral testing for the EPM 30 min after the first extinction session. (**E**) Distance traveled in the whole apparatus. (**F**) Total immobility time. (**G**) Entries and (**H**) time spent in open arms of the maze. (**I**) Entries and (**K**) time spent in closed arms of the maze. All values are presented as mean ± SEM (*n*= 7–8 female mice per condition)

The paradigm utilized for contextual fear conditioning was slightly adjusted from Sanders et al. (2005), to obtain a more gradual learning curve and, in turn, greater temporal resolution for analysis at each stage of the paradigm. Mice were fear-conditioned to a context (Context A) by receiving two inescapable mild electric foot shocks (0.40 mA, 2-s duration, 30 s apart) for 4 consecutive days. Context A consisted of a square Plexiglas chamber (dimensions: 30.5×24.1×21.0 cm), with a bare metal grid floor, no insert along the walls, and no added scent. The chamber is inside a soundproof box developed by Med. Associates. The total length of the conditioning session was 270 s, with a 180-s latency to the first footshock delivery. The mice were transported from the Vanderbilt housing facility to the testing room 30 min before each trial, to allow for acclimation. The extinction training phase of the paradigm consisted of a 600-s exposure to the same context without any footshock, repeated across three consecutive days (Kishimoto et al. 2015), to obtain a near abolishment of the fear response (Matsuda et al. 2015). On day 8, mice were placed in the same context for a single session of 182 s and tested for retrieval of the extinction memory. The nomenclature for the stages of conditioning, extinction training, and retrieval was adopted from Morena et al. (2021). At the end of each session, the mice were placed back in their home cages and later returned to the housing facility. DO34 was administered 2 h before behavioral testing as follows: DO34 before conditioning (Figs. 1, 2, 3, 4, 5, 6); DO34 before extinction training (Figs. 7, 8, 11, 12, 13); DO34 before extinction retrieval (Figs. 9, 10). Based on preliminary data results, the need for three additional experiments as controls emerged, with slight modifications to the described protocol. The first one was a submaximal conditioning protocol, in which the shock intensity was reduced to 0.25 mA. The second one was a delayed protocol, with a delay of 4 days introduced between conditioning and extinction training. During this delay, mice were kept in their home cage without any disturbance. The last control experiment was a sham conditioning with no electric footshock delivery during conditioning sessions. Freezing behavior was measured using video analysis software (Video Freeze-Med Associates). Freezing is defined as the absence of all muscle movements except those required for breathing and it is used as a standard index of conditioned fear (Blanchard and Blanchard 1969).

### Anxiety-like behavior test

To test whether DO34 administration increased general levels of unconditioned anxiety and innate avoidance behavior during extinction, the Elevated Plus Maze (EPM) Test, a well-validated test for anxiety-like behavior in rodents, was performed. Briefly, new mice cohorts were fear-conditioned exactly as before and received DO34 during the extinction phase of the paradigm. After the end of the first extinction session, mice were returned to their homecage for 30 min and then each mouse explored the EPM for 5 min per test; with ANY-maze (Stoelting, Wood Dale, IL) video tracking software used to monitor and analyze behavior during testing. The EPM apparatus had two wall-free open arms (30 × 10 cm; light illuminance ~100-115 lux) and 2 walled (“closed”) arms (30 × 10 × 15 cm; ~20-25 lux), anchored to a square 5 × 5 cm open center. To begin the test and trigger video tracking, mice were placed in the center, facing an open arm. The maze was made of white acrylonitrile butadiene styrene plastic and elevated 50 cm off the ground. The following parameters were analyzed: time and entries to open arms, and time and entries to closed arms. We also checked for total immobility time and distance traveled in the whole apparatus to evaluate changes in global locomotor activity (Dubreucq et al. 2010; Mizuno et al. 2022).

### Statistical analysis

The freezing data for each group was analyzed using a repeated measures two-way ANOVA factoring trial block (time) and drug treatment. For the retrieval session, the whole session freezing readout was averaged and the *P* value was obtained using a two-tailed Student’s *t*-test. For anxiety-like behavior in the EPM, the parameters were analyzed using a two-tailed Student’s *t*-test. Total freezing time and anxiety-like behavior measurements were entered as reported by the video analysis software. All statistical analyses were conducted using Prism GraphPad 9. *P* < 0.05 was considered significant throughout.

## Results

### Pharmacological DAGL inhibition enhances learning of contextual fear conditioning

We first tested whether learning of contextual fear was affected via administration of the DAGL inhibitor DO34 before each conditioning session (days 1–4; Fig. 1A). We found significant increases in freezing only on day 3 of conditioning after DO34 treatment [Fig. 1B: day 1: *P*=0.5490, *F*(1,23)=0.5519; day 2: *P*=0.4709, *F*(1,23)=0.5374; day 3: *P*=0.0349, *F*(1,23)=5.026; day 4: *P*=0.7284, *F*(1,23)=0.1236]. DO34-treated mice showed increased freezing throughout all extinction days (drug-free) [Fig. 1C: day 5: *P*<0.0009, *F*(1,23)=14.60; day 6: *P*<0.0001, *F*(1,23)=32.67; day 7: *P*=0.0220, *F*(1,23)=6.039]. During extinction retrieval (day 8), DO34-treated mice exhibited levels of freezing similar to VEH-treated mice [Fig. 1D: *P*=0.3502; *t*=0.95250, df=23].

We also examined the effects of DO34 on the learning of contextual fear conditioning in female mice. Drug-treated female mice displayed higher levels of freezing compared to VEH subjects during days 3 and 4 of conditioning [Fig. 2B: day 1: *P*=0.8137, *F*(1,22)=0.05689; day 2: *P*=0.9737, *F*(1,22)=0.001116; day 3: *P*=0.0021, *F*(1,22)=12.09; day 4: *P*<0.0001, *F*(1,22)=23.32]. Furthermore, DO34-treated mice exhibited elevated freezing behavior during all three extinction training sessions [Fig. 2C: day 5: *P*=0.0006, *F*(1,22)=15.98; day 6: *P*=0.0014, *F*(1,22)= 13.38; day 7: *P*=0.0290, *F*(1,22)= 5.460]. On extinction retrieval (day 8), there was a significant between-group differences in freezing behavior [Fig. 2D: *P*=0.0121; *t*=2.733, df=22].

To confirm that DO34 was able to increase the learning of contextual fear conditioning, we repeated these experiments using a submaximal conditioning protocol (2 US, 0.25 mA), with DO34 or VEH administration before each conditioning day (Fig. 3A). We found significant increases in freezing on day 4 of conditioning after DO34 treatment in male mice [Fig. 3B: day 1: *P*=0.7968, *F*(1,22)=0.06791; day 2: *P*=0.6319, *F*(1,22)=0.2361; day 3: *P*=0.3034, *F*(1,22)=1.111; day 4: *P*=0.0102, *F*(1,22)=7.894]. The subjects also exhibited sustained freezing for all extinction training days (drug-free) [Fig. 3C: day 5: *P*=0.0047, *F*(1,22)=9.867; day 6: *P*=0.0006, *F*(1,22)=15.83; day 7: *P*=0.0046, *F*(1,22)=9.974]. During extinction retrieval (day 8), DO34-treated mice exhibited levels of freezing similar to VEH-treated mice [Fig. 3D: *P*=0.1000; *t*=1.717, df=22]. In comparison, female mice displayed higher levels of freezing compared to VEH subjects during days 3 and 4 of conditioning [Fig. 4B: day 1: *P*=0.6105, *F*(1,12)=0.2735; day 2: *P*=0.8837, *F*(1,12)=0.02234; day 3: *P*=0.0256, *F*(1,12)=6.484; day 4: *P*=0.0050, *F*(1,12)=11.73]. Similarly to male mice, female mice also exhibited elevated freezing behavior throughout extinction training sessions [Fig. 4C: day 5: *P*=0.0332, *F*(1,12)=5.786; day 6: *P*=0.0266, *F*(1,12)=6.384; day 7: *P*=0.0049, *F*(1,12)= 11.83]. When tested for extinction retrieval (day 8), there were no significant between-group differences in freezing behavior [Fig. 4D: P=0.6795; *t*=0.4234, df=12].

We next conducted a series of experiments exactly as described above, but with a 4-day delay between conditioning and extinction (Fig. 5A). We found that male mice treated with DO34 during conditioning froze significantly more than VEH-treated mice during 2 out of 4 conditioning sessions [Fig. 5B: day 1: *P*=0.1264, *F*(1,18)=2.569; day 2: *P*=0.0037, *F*(1,18)=11.13; day 3: *P*=0.0347, *F*(1,18)=5.218; day 4: *P*=0.4253, *F*(1,18)=0.6655]. However, mice did not exhibit significant differences in freezing behavior for any of the extinction days after a 4-day delay [Fig. 5C: day 9: *P*=0.2716, *F*(1,18)=1.286; day 10: *P*=0.9045, *F*(1,18)=0.01480; day 11: *P*=0.1773, *F*(1,18)=1.971]. Furthermore, both treatment groups exhibited similar levels of freezing during extinction recall on day 12 (Fig. 5D: *P*=0.3987; *t*=0.8644, df=18).

Regarding female mice, DO34-treated mice froze significantly more than VEH-treated mice during 2 out of 4 conditioning sessions [Fig. 6B: day 1: *P*=0.9617, *F*(1,13)=0.002392; day 2: *P*=0.2648, *F*(1,13)=1.358; day 3: *P*=0.0001, *F*(1,13)=28.61; day 4: *P*=0.0014, *F*(1,13)=16.45]. Mice treated with DO34 during conditioning did not exhibit significant differences in freezing behavior for any of the extinction days [Fig. 6C: day 9: *P*=0.3829, *F(*1,13)=0.8156; day 10: *P*=0.5485, *F*(1,13)=0.3796; day 11: *P*=0.1859, *F*(1,13)=1.950]. However, during extinction retrieval on day 12, female mice that received DO34 treatment during conditioning displayed lower freezing levels (Fig. 6D: *P*=0.0007; *t*=4.563, df=13).

### Pharmacological DAGL inhibition impairs extinction of contextual fear conditioning

To test whether learning of fear extinction is affected by the depletion of 2-AG levels, DO34 was administered to male and female subjects during the extinction training phase of our paradigm. We observed significant increases in freezing behavior in DO34-treated compared to VEH-treated male mice during each extinction session [Fig. 7B: day 5: *P*=0.002, *F*(1,18)=20.79; day 6: *P*=0.0004, *F*(1,18)=19.18; day 7: *P*=0.0102, *F*(1,18)=8.232]. During extinction retrieval (day 8), DO34-treated mice did not display differences in freezing levels (Fig. 7D: *P*=0.1571; *t*=1.476, df=18). Female mice treated with DO34 exhibited increased freezing levels during 2 out of 3 extinction sessions [Fig. 8C: day 5: *P*=0.6223, *F*(1,18)=0.2512; day 6: *P*=0.0454, *F*(1,18)=4.623; day 7: *P*=0.0074, *F*(1,18)=9.097]. Finally, DO34- and VEH-treated female mice showed no significant differences in freezing behavior [Fig. 8D: *P*=0.0962; *t*=1.756, df=18] when tested for extinction retrieval (day 8).

### Pharmacological DAGL inhibition does not impair retrieval of learned extinction

For the last set of experiments, we aimed to determine whether 2-AG signaling is required for retrieval of the extinction learning (Fig. 9A). During conditioning, male mice displayed elevated freezing levels only during day 1 [Fig. 9B: *P*=0.0462; *F*(1,18)= 4.583]. Upon allocation to either the DO34 or VEH treatment group after matching both the conditioning and extinction learning curves (Fig. 9B, C), results showed no significant differences in freezing behavior between DO34 and VEH-treated male mice [Fig. 9D: *P*=0.9430; *t*=0.07255, df=18] during extinction retrieval (day 8).

The same experiment was conducted but using female mice (Fig. 10A–D). Data obtained revealed no significant differences in freezing behavior [Fig. 10D: *P*=0.5471; *t*=0.6144, df=17] between treatment groups for female mice.

### Pharmacological DAGL inhibition increases unconditioned freezing

To test the possibility that DO34 augments freezing behavior independent of fear conditioning, we performed a sham-conditioning protocol and subjected mice to DO34 administration before sham extinction sessions. Results showed that for male mice, behavior was not different between experimental subjects during any of the sham-conditioning sessions [Fig. 11B: day 1: *P*=0.5324, *F*(1,21)=0.4030; day 2: *P*=0.4468, *F*(1,21)=0.6010; day 3: *P*=0.7324, *F*(1,21)=0.1201; day 4: *P*=0.6460, *F*(1,21)=0.2172]. Drug treatment during extinction training (Fig. 11C) did not heighten freezing behavior on day 5 [Fig. 7C: *P*=0.3183, *F*(1,21)=1.045]. However, repeated DO34 administration slightly, but significantly, elevated unconditioned freezing levels on day 6 [*P*=0.0072, *F*(1,21)=8.877] and day 7 [*P*<0.0001, *F*(1,21)=27.20]. For female mice, the behavior between DO34- and VEH-treated subjects was only different during the first sham-conditioning session [Fig. 11E: day 1: *P*=0.0339, *F*(1,13)=5.617; day 2: *P*=0.4337, *F*(1,13)=0.6526; day 3: *P*=0.7654, *F*(1,13)=0.09282; day 4: *P*=0.4780, *F*(1,13)=0.5337]. Drug treatment during extinction (Fig. 11F) slightly heightened freezing behavior only on day 7 [Fig. 11F: day 5: *P*=0.4187, *F*(1,13)=0.6976; day 6: *P*=0.4207, *F*(1,13)=0.6914; day 7: *P*=0.0085, *F*(1,13)=9.591].

### Pharmacological DAGL inhibition does not affect innate avoidance behavior

As a final measure of whether DO34 administration increases unconditioned anxiety and avoidance behavior, a new cohort of male mice was tested for anxiety-like behavior in the Elevated Plus Maze 30 min after their first extinction session with DO34 or VEH treatment (Fig. 12D). The freezing levels for each stage of the fear conditioning and extinction paradigm are shown in Fig. 12A–C, replicating our earlier data in which DO34-treated subjects exhibit higher freezing levels. However, results revealed no significant differences in time spent nor entries in open vs. closed arms between DO34 and VEH-treated male mice (Fig. 10G–J; *P*>0.05). A similar scenario was observed when analyzing the distance traveled and time immobile in the apparatus during the whole session (Fig. 12E, F). On the other hand, female mice demonstrated higher freezing levels only during the second extinction session [Fig. 13B: day 6: *P*=0.0489, *F*(1,13)=4.719). However, no differences were found in the distance traveled nor in time immobile in the apparatus during the whole session (Fig. 13E, F). VEH-treated female mice had more entries to the closed arms of the EPM, in comparison to DO34-treated subjects (Fig. 13H: *P*=0.0340; *t*=2.370, df=13).

## Discussion

Previous studies have implicated 2-AG signaling in stress adaptation and modulation of anxiety-like behavior after stress exposure (Bedse et al. 2017; Bluett et al. 2017; Marcus et al. 2020; Patel et al. 2005; Patel and Hillard 2008; Patel et al. 2009). However, a comprehensive examination of 2-AG signaling depletion in contextual fear conditioning is not available and thus addressed by the current study. Upon DAGL inhibition during contextual fear conditioning, we found that male and female mice showed enhanced fear learning as reflected by a progressive increase in freezing across conditioning days relative to vehicle treatment in our conventional and subthreshold conditioning protocols, an effect that was more pronounced in female mice. Whether these effects are due to enhanced acquisition or consolidation is not clear from the current protocol. These data suggest endogenous 2-AG signaling acts to suppress the learning of contextual fear memories.

Interestingly, after DO34 treatment during conditioning, we observed impaired within-session extinction in the drug-free state. There can be several explanations for this effect. The first is that DO34 may strengthen the context-US association, as indicated by enhanced freezing during conditioning using our conventional and submaximal conditioning, which could result in extinction impairment in the drug-free state. However, the lack of within-session extinction impairment in our experiments which contained a 4-day delay between conditioning and extinction suggests this is not the case. Another possibility is that enhanced fear learning observed after DO34 treatment during conditioning could render memories more resistant to extinction only within a short time interval (i.e., days 5–7) since dynamics of recent versus remote aversive fear memories are different and can undergo circuit reorganization with time (Do-Monte et al. 2015; Frankland et al. 2004; Santini et al. 2001). It is also possible that DO34 administration increases sensitized freezing to context, which then manifests as an impairment in the within-session learning of extinction. Sensitization is a non-associative learning process, which is characterized by enhanced responsiveness to potentially harmful stimuli after stress exposure (Lutz 2007). That adding a delay between the exposure to aversive stimuli (conditioning) and re-exposure to the context in the form of extinction training eliminated the within-session extinction impairment is consistent with this hypothesis, as sensitization is time-dependent. Experiments by Kamprath and Wotjak (2004) showed that non-associative learning processes such as sensitization and habituation can influence the extinction of conditioned fear in mice (Kamprath and Wotjak 2004). Furthermore, we and others have previously proposed a role for 2-AG in the regulation of sensitization and habituation (Patel and Hillard 2008; Kamprath et al. 2006; Plendl and Wotjak 2010). A minor concern would be the possible carry-over effects of the drug onto extinction days due to repeated drug administration during conditioning. On that note, DO34 is an irreversible inhibitor with an approximate half-life of 4–6 h like most triazole ureas, but 2-AG levels after 24 h appear to be still lower than in vehicle-treated mice (Ogasawara et al. 2016). Therefore, although a possible carry-over effect might have influenced the first day of extinction, we do not think this explains the elevated freezing behavior during sessions 2 and 3 of extinction.

Both male and female mice treated with DO34 during extinction training showed impairments in the within-session learning of contextual fear extinction. Relevant to this finding, we and others have previously suggested a role for 2-AG signaling in the extinction of cued conditioned fear responses. Specifically, mice lacking DAGLα- and DO34-treated mice both show impaired extinction in auditory-cued fear conditioning paradigms (Cavener et al. 2018; Jenniches et al. 2016). These data agree with previous literature reporting an important role for CB1 receptors in the modulation of fear extinction (Hammoud et al. 2019; Lin et al. 2009; Marsicano et al. 2002; Pamplona et al. 2006; Rabinak et al. 2014; Rabinak et al. 2013). In contrast to these studies, there are also reports of impairments in extinction learning with *increased* levels of 2-AG, via pharmacological (Hartley et al. 2016; Mizuno et al. 2022) or genetic (Kishimoto et al. 2015) inhibition of MAGL. One explanation for this apparent contradiction is based on the well-known bi-phasic effects of CB1 activation on fear and anxiety responses, with low doses generally reducing anxiety and fear, while high doses potentiate these responses. Genetic studies have suggested the anxiogenic effects of high doses of cannabinoids are mediated via activation of CB1 on GABAergic neurons, while the low-dose anxiolytic effects of cannabinoid are mediated via activation of CB1 on glutamatergic terminals (Rey et al. 2012). Based on these data, Llorente-Berzal et al. have suggested excess 2-AG generated via MAGL inhibition may preferentially engage CB1 on GABA synapses in an ectopic fashion promoting fear and impairing extinction (Llorente-Berzal et al. 2015). If correct, a corollary of this hypothesis is that DO34-induced depletion of 2-AG impairs physiological actions at CB1 receptors on forebrain glutamatergic neurons which reduce anxiety and fear responses. Genetic studies in GABA and glutamate-specific CB1 knock-out mice will be needed to formally test this hypothesis in a contextual fear conditioning paradigm. Lastly, we did not see any prominent increases in avoidance behavior in the EPM on day 1 of extinction in DO34-treated mice, suggesting the effects on extinction impairment may be independent of unconditioned avoidance behavior.

After extinction training, mice were assessed for retrieval of the extinction memory on day 8 in the drug-free state, in which both male and female DO34- and VEH-treated mice exhibited similar levels of freezing. Therefore, the retrieval of extinction training appears to be unaffected by DO34 treatment during extinction training. These results indicate the critical role of 2-AG in the regulation of freezing behavior and within-session learning of extinction. Although the importance of within-session extinction for the overall learning of extinction has been debated, it is dependent on CB1R signaling (Plendl and Wotjak 2010). Furthermore, there is evidence that within-session extinction correlates with rates of fear relapse (King et al. 2018). Lastly, DO34 treatment before extinction retrieval did not affect freezing for either male or female mice. This suggests no role for 2-AG in the retrieval of the extinction memory, once it has been successfully learned.

It is noteworthy that DO34 causes a small but significant increase in unconditioned freezing after repeated treatment. This is consistent with the anxiogenic effects of pharmacological and genetic DAGL inhibition (Bluett et al. 2017; Jenniches et al. 2016; Shonesy et al. 2014). However, this effect was only seen after 3 days of injections and thus cannot fully explain within-session extinction deficits which are seen on the first day of DO34 treatment in our contextual fear conditioning paradigm. How 2-AG may regulate learned and innate freezing behavior differentially remains to be determined at the mechanistic level.

It is worth mentioning that males and females can respond differently to drugs or genetic manipulations that act on 2-AG signaling or CB1 receptors, respectively, during anxiety-like behavior tests (Salemme et al. 2023; Bowers and Ressler 2016); however, both male and female mice showed qualitatively similar responses to DO34 in our studies and previous experiments using cued conditioning paradigm (Cavener et al. 2018). Although sex differences have been reported previously for context fear conditioning (Colon and Poulos 2020; Fenton et al. 2016; Keiser et al. 2017; Maren et al. 1994; Russo and Parsons 2021; Trott et al. 2022) or extinction (Baran et al. 2010; Daviu et al. 2014; Matsuda et al. 2015; Morena et al. 2021), other studies have demonstrated a lack of sex differences (Gruene et al. 2015; Mizuno et al. 2022; Urien et al. 2021). Moreover, no consistent sex differences have been found regarding 2-AG levels (Morena et al. 2021; Vecchiarelli et al. 2022) in corticolimbic areas involved in fear learning and extinction across different stress modalities. The only noteworthy difference was found by Levine et al. (2021), who reported that basal 2-AG concentration in the PAG was lower in female than male rats; an effect demonstrated to not be dependent on estrous stage (Levine et al. 2021).

Limitations of the study include sole reliance on pharmacological DAGL inhibition. Previous studies have demonstrated DAGLα knock-out mice show extinction impairment (Cavener et al. 2018; Jenniches et al. 2016), providing confidence that our current data is not confounded by off-target drug effects. Regarding data interpretation, the timeline of DO34 administration in our experimental paradigm does not allow us to distinguish between acquisition and consolidation processes during conditioning or extinction training. Furthermore, it is known that DAGL inhibition alters levels of several monoacylglycerols and can deplete 2-AG degradation products including free arachidonic acid (Ogasawara et al. 2016; Wilkerson et al. 2017). While previous data heavily impacting CB1 receptors—a primary receptor target of 2-AG—in fear extinction strongly suggests the behavioral effects of DAGL inhibition seen here are likely due to 2-AG deficiency, effects of other lipid alternations on the behavioral phenotypes cannot be excluded. Another minor limitation could be the fact that only one dose of DO34 was used throughout the experiments. However, this is the standard dose used in our lab across many behavioral experiments to successfully elicit a near depletion of 2-AG levels in the brain in general (Ogasawara et al. 2016) and also in specific regions of the fear circuitry (Bluett et al. 2017).

Although not everyone who experiences or witnesses trauma develops a trauma or stressor-related disorder, these disorders seriously undermine the mental health of affected individuals (Baxter et al. 2014; Kessler 2007) and represent a public health challenge (Maren and Holmes 2016). Enhanced contextual fear learning observed after DO34 treatment is consistent with a large volume of clinical data indicating 2-AG deficient states worsen behavioral consequences of stress exposure (Crombie et al. 2019; Fitzgerald et al. 2021; Hill et al. 2013; Ogasawara et al. 2016; Spohrs et al. 2022; Yi et al. 2016). These data suggest that lower 2-AG levels could represent a susceptibility endophenotype supporting aversive contextual associative learning *and* preventing the extinction of aversive memories, potentially predisposing to trauma-related disorders. The degree to which individual differences in 2-AG levels before trauma contribute to PTSD susceptibility is not known. In addition, our data support 2-AG signaling as a prominent regulator of within-session contextual fear extinction in male and female mice, potentially due to associative and non-associative learning processes.
